# Adult neurogenesis and its promise as a hope for brain repair

**DOI:** 10.3389/fnins.2014.00165

**Published:** 2014-06-17

**Authors:** Carlos Lois, Wolfgang Kelsch

**Affiliations:** ^1^Department of Neurobiology, University of Massachusetts Medical SchoolWorcester, MA, USA; ^2^Department of Psychiatry and Psychotherapy, Central Institute of Mental Health, University of HeidelbergMannheim, Germany

**Keywords:** neurogenesis, synapses, brain repair, regeneration, brain circuitry

After the pioneer report by Joseph Altman of adult neurogenesis (AN) in mammals in 1962, the phenomenon of AN was “rediscovered” some 20 years later, first in songbirds and then in mammals. Since the 1990s, interest in AN was fueled by the hope that it could lead to the treatment of neurological deficits by grafting these neurons or their progenitors into brain areas affected by disease or injury. Unfortunately, after 20 years of intense research efforts there is no clear indication that AN can be harnessed for the repair of brain circuits. We argue that the exuberant optimism regarding the potential application of AN for brain repair was misguided by the belief that neurons and their precursors had extensive developmental plasticity. Many of the experiments investigating the potential of AN for brain repair were inspired by the idea that neuronal precursors would be able to adapt, and easily change their developmental fate to replace the lost neurons. However, research during the last 20 years has shown that, in most cases, the fate of neurons is strongly determined and that it rarely changes. Understanding the mechanisms that control neural cell fate may allow for the engineering of adult stem cells so that they can give rise to neurons with properties appropriate for the host circuit to be repaired. The lack of phenotypic flexibility of neuronal progenitors may eventually prove to be advantageous, as this may provide a high degree of predictability (and safety) in the properties of reprogrammed cells. We suggest that AN is still a useful model to understand how neurons integrate into adult brain circuits, and that brain repair will require a thorough understanding of the genetic programs that control neuronal fate and neuronal migration.

A cut in the skin is repaired within a few days, and a broken bone heals in a few weeks. In contrast, damage to the nervous system results in deficits that are only partially reversible. This limited ability for functional recovery led to the widely held belief that the brain and spinal cord are not able to regenerate. A direct challenge to this assumption was launched by the pioneer discovery by Joseph Altman who first described neurogenesis in the brain of adult rats (Altman, 1962). Using radioactively-tagged thymidine, Altman suggested that new neurons were added into several regions of the adult rat brain, including into the olfactory bulb (OB) and dentate gyrus (DG) (Altman and Das, 1965).

The initial discovery of Altman was mostly ignored for two decades, until Goldman and Nottebohm reported neurogenesis in the brain of adult canaries (Goldman and Nottebohm, 1983). However, the impact of this discovery was also limited, because many considered AN in birds an oddity that could not occur in mammals. So, despite a few occasional studies confirming the addition of new neurons into the brain of adult mammals in the 1970s and 1980s, the phenomenon of mammalian AN was outside of mainstream neuroscience for many years.

Interest in mammalian brain repair and AN gained momentum in the early 1990' with the discovery that the adult mouse brain contained stem cells that could be induced to proliferate *in vitro* by the addition of growth factors (Reynolds and Weiss, 1992). These stem cells grew to form aggregates of cells called “neurospheres” and differentiated into neurons and glia. The discovery of neurospheres galvanized the field of brain repair and for the first time provided researchers with a robust *in vitro* system to produce new neurons from adult mammalian brains in large quantities and revitalized the interest in neuronal transplantation as a brain repair strategy. The goal of neuronal transplantation is to add neurons into the brain to repair brain lesions. In AN, new neurons are spontaneously added into the functioning circuits of a mature brain. Thus, neuronal transplantation and AN inform each other reciprocally. Grafting different cell types into the brain provided novel insights into the factors regulating survival and integration of new neurons into brain circuits (Stenevi et al., 1976). Moreover, the techniques of neuronal transplantation enabled some key findings in AN. However, despite this progress, the clinical applications of grafting neurons have not materialized yet, and remains an experimental method for research.

In parallel with the progress in neuronal stem cell research and neuronal transplantation, important advances were made on the question of neurogenesis in the adult mammalian brain. In the early 1990s it was confirmed that there were two main areas of the rodent brain that received new neurons, the OB and the hippocampal DG. The progenitors of new neurons are located in the subventricular zone (SVZ) and in the subgranular zone (SGZ) of the DG. The neuroblasts generated in the SVZ migrate long distances to reach the adult OB and differentiate into neurons (Lois and Alvarez-Buylla, 1994), suggesting that grafted neurons or progenitors could also have the potential to migrate throughout the brain. However, it was later found that only a specific type of neuronal progenitors derived from the medial ganglionic evidence (MGE) had the ability to disperse broadly (Wichterle et al., 1999).

Interestingly, the original reports by Altman suggested that new neurons may be added to brain regions such as the cerebral cortex and thalamus (Altman, 1962). The late 1990s saw a resurgence in the possibility that neurons are added outside of the OB and DG (for example into the neocortex, striatum or hippocampal CA1), both spontaneously or after lesions. Many of these reports have been surrounded by controversy, and the generally accepted view is that AN is mostly limited to the OB and DG. The possibility remains that low levels of AN exist in other brain regions (Ernst et al., 2014) and that under certain circumstances young neurons could be recruited into regions outside of the OB and DG.

The spectacular advances in animal cloning in the late 1990s, pioneered by the generation of Dolly the sheep, gave new impetus to the question of cellular phenotypic flexibility (Wilmut et al., 1997). If a fibroblast form the mammary gland could be reprogrammed to produce a whole sheep, perhaps a neuronal stem cell could be reprogrammed to produce any lost neuron in the brain. In the late 1990s several studies suggested that transplanted stem cells have great phenotypic flexibility. For example, it was reported that blood stem cells appeared to have the potential to become neurons in the brain. Most of these cases of “transdifferentiation” were likely due to fusion of the grafted cells and resident cells. Recent experiments rather indicate that neuronal stem cells are predetermined to generate specific neuronal types, and that altering the environment in which those progenitors differentiate does not change the type of neurons that they produce (Kelsch et al., 2007; Merkle et al., 2007).

The perspective gained from these last 20 years suggests that adult mammalian neurogenesis is mostly confined to the OB and DG, and that neuronal progenitors appear to have very limited (if any) phenotypic flexibility. In view of these constraints, it seems clear why the initial hopes for the therapeutic potentials of AN have not materialized. Next, we would like to suggest strategies so that AN could be harnessed for brain repair.

## Prospects of adult neurogenesis for brain repair: suggestions

There are two main types of neurons produced in the mammalian brain during AN: granule neurons in the OB and the DG. In addition, other neuronal types are also produced in the OB and the DG, but in much smaller numbers (such as periglomerular cells for the OB).

Adult neurogenesis may be useful to replace the granule cells in the OB and DG in situations where these cells are lost to injury or disease. However, it is unlikely that grafting these cells or their progenitors could have any beneficial effects in any other brain regions, for two main reasons: First, transplantation of these cells leads to a mass of clumped cells at the graft site, and the vast majority of cells fail to migrate to colonize the surrounding parenchyma. If cells cannot disperse through the brain, they cannot reach the sites where they are needed, and thus, they cannot replace the neurons lost to disease or injury. Second, the proper function of brain circuits requires that neurons with defined properties perform specific functions. Both adult-born granule cells in the OB and DG have highly specialized properties. There is no evidence that grafting of progenitors can lead to a spontaneous reprogramming of their phenotypes. Thus, it is difficult to imagine how grafting cells with predetermined properties tailored for the function of a highly specialized circuit may lead to functional restoration of a completely different circuit in another part of the brain.

In summary, we believe that the therapeutic potential of the endogenous, unmanipulated neuronal progenitors in the mammalian brain may not be promising. At the same time, we believe that AN offers an outstanding opportunity to learn principles about how new neurons integrate into mature brain circuits, and that these principles can guide the use of engineered stem cells for brain repair.

We would like to offer some specific suggestions that we believe could be useful for the design of therapeutic strategies for brain repair:

***Determination of cell fate:*** The appropriate function of a circuit requires neurons with specialized properties and defined patterns of connections. To maximize the chances that a new neuron will restore the function of a damaged circuit, it will be crucial to define the mechanisms by which a stem cell can be directed to a specific fate so that the new neuron has properties as similar as possible as those of the lost neurons. Identifying the genetic programs that control neuronal differentiation will eventually allow for the generation of neuronal populations with well-defined identity. However, for some applications the requirements for specific neuronal identity may not be so crucial. For example, for neurological disorders due to hyperactivity (e.g., epilepsy), the addition of inhibitory neurons that could simply reduce the overall levels of activity could be sufficient to ameliorate the outcome of the disease.
***Migratory ability:*** One of the main hurdles for neuronal replacement therapies is due to the very limited ability of grafted neurons to migrate through the adult brain so that they can reach the sites where neurons have been lost. Currently, the only neuronal progenitors that can migrate extensively through the adult brain are those from the embryonic medial ganglionic eminence (MGE). Unfortunately, the MGE progenitors only give rise to inhibitory interneurons. Transplanting interneurons could be a useful intervention to control neurological diseases characterized by hyperactivity, such as epilepsy. However, it is unlikely that transplanting MGE progenitors could restore function in the most common neurological disorders, such as Alzheimer's, Parkinson's or stroke, which are characterized by neuronal loss. There are 2 avenues that would be worth exploring regarding these issues. First, we should investigate the molecular mechanisms that enable MGE progenitors to disperse widely through the brain. Perhaps delivering the genes that confer the migratory ability of MGE cells into neuronal stem cells for other cell types (e.g., stem cells for projection neurons) would enable them to disperse through the brain. Second, MGE cells could be engineered so that they retain their migratory ability, but can be tailored to switch their cell fate, and thus, they could give rise to other neuronal types after they complete their migration.
***Integration into brain circuits:*** After young neurons have completed their migration they have to form appropriate synaptic connections with the pre-existing neurons. Perhaps this could the most challenging of all aspects of brain repair. The mechanisms that control synapse formation, selection, and maintenance are only partially understood, and these are highly complex processes. Moreover, after brain lesions new neurons will face damaged circuits, thus increasing the difficulty of making the correct synaptic connections. Again, AN could offer crucial clues about how this process could be recapitulated with grafted neuronal progenitors/stem cells. Two areas of research could be fruitful to enable the appropriate wiring of young neurons into adult circuits.
  - We still do not have a good understanding of the role of electrical activity on the formation and maintenance of synapses as new neurons integrate into brain circuits during AN. For example, it is not clear what are the respective roles of the activity of the pre-existing neurons vs. the cell-autonomous activity of the new neurons on the integration of the young neurons (Lin et al., 2010). Investigating these issues could offer important clues to design strategies to optimize the integration of grafted young neurons into the brain.
  - Second, identifying molecular pathways that regulate the formation of specific synapses between neurons could lead to a strategy where these molecules are expressed in the young neurons to “force” the formation of synapses.

Fifty years after the discovery of AN the field is realizing that some of the initial hopes to use this process for brain repair were naively optimistic. The assembly of brain circuits is an extremely complicated process, and it is not realistic to hope that young neurons would “know what to do” when faced with a damaged brain to restore its structure and function. Dispersion through the adult brain is a major bottleneck that has to be resolved to achieve efficient brain repair. We now know that there are strong genetic determinants that define the identity of the progeny produced by neuronal progenitors, and that young neurons (endogenous or grafted) do not have the ability to “adapt” their identities to the needs of the circuit (Kelsch et al., 2007; Merkle et al., 2007). Finally, we need to understand the principles that guide the formation of synapses between young and pre-existing neurons to enable the appropriate wiring of the circuit. Using neuronal replacement to achieve brain appears a daunting prospect, but we know that addition of new neurons into pre-existing circuits is exactly what happens during AN. Adult-born neurons may not be useful to repair the brain, but learning from AN should guide our attempts to use engineered stem cells to achieve this goal.

### Conflict of interest statement

The authors declare that the research was conducted in the absence of any commercial or financial relationships that could be construed as a potential conflict of interest.
